# LBBB: State-of-the-Art Criteria

**Published:** 2013-06-01

**Authors:** Mohammad Hosein Nikoo, Amir Aslani, Mohammad Vahid Jorat

**Affiliations:** 1 Cardiovascular Research Center, Shiraz University of Medical Sciences, Shiraz, IR Iran

**Keywords:** Left Bundle Branch Block, Criteria

We started diagnosing Bundle Branch Block about 100 years ago on dog models (1). However, about 40 years passed until we could diagnose Left Bundle Branch Block (LBBB) correctly on ECG (2).

Today, we have conventional criteria for diagnosing LBBB, including QRS duration>120 msec, QS or rS in lead V1, Monophasic R wave with no Q wave in lead V6 and I3, ACC/AHA/HRS added notched R wave in lead I,aVL, V5, and V6, and occasional RS pattern in V5 and V6 (3).

In case rate dependent LBBB develops, you can see the disappearance of the q wave in V6, then initial slurring of R wave and delayed increased intrinsicoid deflection. More complete LBBB causes notched plateau after initial peaked R wave (4).

LBB has anterior fascicle, posterior fascicle, and sometimes a septal fascicle (5).

Blocking the left bundle may cause septal force to disappear; therefore, no initial R wave can be detected in V1 or Q wave in I, V5, and V6, but that is not always the case (5).

Sometimes, septal MI causes initial Q in the lateral leads and initial R wave in V1. 

Also, Grants and Doge found initial septal force in 40% of their cases with LBBB (6).

Accordingly, initial septal force should not be considered as a diagnostic criterion for LBBB. Widening of QRS may occur in LBBB as well as many other conditions, such as LVH, RVH, and IVCD. Sometimes, LBBB also causes minimally increased width in QRS named incomplete LBBB.

Wilson compared dogs and humans and suggested 120 msec. as the cut-off point for diagnosing LBB (2); however, this may need revision based on the findings of the study by Selvester and Salmon (7).

They showed that when LBB is blocked, 40 msec. are required for septal depolarization, then 50 msec to reach the posterolateral wall, and finally 50 msec to complete posterolateral wall activations. Moreover, they suggested 140 msec. for males and 130 msec. for females for diagnosis of LBBB. The most consistent finding in LBBB patients seems to be mid QRS notching or slurring which is best seen in I, aVl, V5, and V6 (3).

This mid QRS notching shows two vectors that are in the relatively same direction but one is minimally delayed. The first vector shows depolarization of endocardium of the left ventricle, while the second one seem to show depolarization of epicardium of the posterolateral wall (8).

Diagnosis of LBBB using ECG may be accompanied by some errors as high as 30 % of cases. Therefore, LBBB is better to be confirmed through intracardiac mapping techniques. However, only a limited number of studies have investigated the issue. Josephson nicely mapped about 40 patients and his consistent finding was nearly 40-msec delay between RV endocardium and LV endocardium in LBBB patients (9).

Furthermore, Vassallo et al. showed that only ⅔ of the LBBB patient diagnosed on ECG had more than 40 msec. trans-septal activation on intracardiac mapping; thus, the accuracy of the routine criteria for diagnosis of LBBB was only 70% (10).

In 2004, Auricchio performed 3-dimensional contact and noncontact mapping for LBBB patient and his results showed the same accuracy as the conventional ECG criteria (11).

All these lead us to wrong diagnosis of LBBB in ⅓ of our patients and this is the exact number of non-responders in CRT patients where diagnosis of LBBB is a pre- requisites (12).

Considering what was mentioned above, new criteria for LBBB are needed and the best suggestions include:

QRS more than 140 msec for males and 130 msec for females

Notching of peak QRS in at least two leads from I,avL, V1, V2, V5, and V6

QS or rS in lead V1 (13).

This new definition can be used for picking up the cases of CRT implantation and the patients follow up may solve the mystery of non-responders in CRT patients.
